# Shaping of the nephron – a complex, vulnerable, and poorly explored backdrop for noxae impairing nephrogenesis in the fetal human kidney

**DOI:** 10.1186/s40348-020-0094-9

**Published:** 2020-01-22

**Authors:** Will W. Minuth

**Affiliations:** Institute of Anatomy, University of Regensburg, D-93053 Regensburg, Germany

**Keywords:** Impaired nephrogenesis, Fetal human kidney, Nephrogenic zone, Nephron, Shaping

## Abstract

**Background:**

The impairment of nephrogenesis is caused by noxae, all of which are significantly different in molecular composition. These can cause an early termination of nephron development in preterm and low birth weight babies resulting in oligonephropathy. For the fetal human kidney, there was no negative effect reported on the early stages of nephron anlage such as the niche, pretubular aggregate, renal vesicle, or comma-shaped body. In contrast, pathological alterations were identified on subsequently developing S-shaped bodies and glomeruli. While the atypical glomeruli were closely analyzed, the S-shaped bodies and the pre-stages received little attention even though passing the process of nephron shaping. Since micrographs and an explanation about this substantial developmental period were missing, the shaping of the nephron in the fetal human kidney during the phase of late gestation was recorded from a microanatomical point of view.

**Results:**

The nephron shaping starts with the primitive renal vesicle, which is still part of the pretubular aggregate at this point. Then, during extension of the renal vesicle, a complex separation is observed. The medial part of its distal pole is fixed on the collecting duct ampulla, while the lateral part remains connected with the pretubular aggregate via a progenitor cell strand. A final separation occurs, when the extended renal vesicle develops into the comma-shaped body. Henceforth, internal epithelial folding generates the tubule and glomerulus anlagen. Arising clefts at the medial and lateral aspect indicate an asymmetrical expansion of the S-shaped body. This leads to development of the glomerulus at the proximal pole, whereas in the center and at the distal pole, it results in elongation of the tubule segments.

**Conclusions:**

The present investigation deals with the shaping of the nephron in the fetal human kidney. In this important developmental phase, the positioning, orientation, and folding of the nephron occur. The demonstration of previously unknown morphological details supports the search for traces left by the impairment of nephrogenesis, enables to refine the assessment in molecular pathology, and provides input for the design of therapeutic concepts prolonging nephrogenesis.

## Background

### Clinical aspects

The impairment of nephrogenesis is caused by intra- and extrauterine noxae, which can lead to an early termination of nephron formation in preterm and low birth weight babies [1, 2]. The subsequent oligonephropathy/oligonephronia is estimated to affect between 8 and 24% of babies [3]. The related consequences for health in later life were described in detail [4–7].

The noxae impairing nephrogenesis are quite different in molecular composition and comprise restricted nutrition, particular protein or micronutrient intake, poor antenatal perfusion with lack of oxygen, inflammatory cytokines, reactive oxygen species, and antiangiogenic factors [8–11]. An ambiguous role is played by drugs administered to preterm and low birth weight babies. The therapeutic benefit of drugs is balanced against the frequently unknown nephrotoxic potential [12–15].

### Traces left by the impairment

The traces left by the impairment of nephrogenesis were investigated in the rodent, baboon, and fetal human kidney (Table 1). Previous literature indicates that the impairment is restricted to the outer cortex of the fetal kidney and surprisingly depends on species and focuses at the stages of nephron anlage.

**Table 1:**
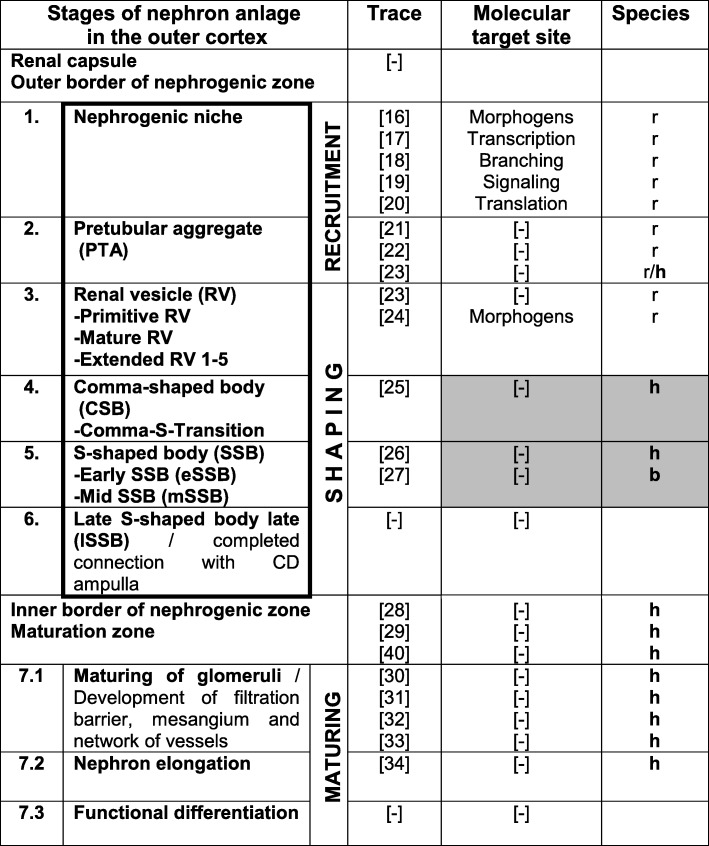
Allocation of nephron formation in the nephrogenic and maturation zones in the outer cortex of the mammalian kidney during the phase of late gestation

The initial development of a nephron occurs in a nephrogenic compartment (framed). The recruitment of progenitor cells takes place in the nephrogenic niche and pretubular aggregate. The shaping of the nephron runs in the renal vesicle, comma-shaped, and S-shaped body. Traces left by damage or scheduled reduction are indicated in gray. Traces were reported from the human **h**, baboon **b**, and rodent r kidney. [**–**] not investigated

#### The nephrogenic niche (Table 1 (no. 1))

A lack of maternal food in rats leads to increased levels of mRNA for morphogens WT1, FGF2, and BMP7, while mRNA for Pax2, GDNF, FGF7, BMP4, Wnt4, and Wnt11 declines [16]. The cessation of nephrogenesis in the mouse changes gen activity in nephrogenic progenitor cells, lowers their number, and inhibits their proliferation [17]. Maternal nutrient restriction in rat reduces ureteric bud branching up to 50%, but surprisingly does not influence the duration of nephron formation [18]. A low protein diet alters the activity of gens in the mouse kidney by decreasing Slit2/Robo2 and Spry1, but stimulating Ret [19]. Maternal malnutrition during pregnancy stimulates the expression of fetuin-B in the mouse kidney, which in turn limits nephron formation by reducing Six2^+^ progenitor cells [20].

#### The pretubular aggregate (Table 1 (no. 2))

In the mouse kidney, morphogen Wnt4 controls after induction the condensation of mesenchymal progenitor cell [21, 22]. In the fetal human kidney, it was shown that the pretubular aggregate stays connected with overlying nephrogenic mesenchymal progenitor cells [23]. A concrete damage on the pretubular aggregate was not reported.

#### The renal vesicle (Table 1 (no. 3))

Malnutrition in mouse neonates leads to 76% less Six2^+^ nephrogenic progenitor cells and decreases the expression of morphogens Wnt9b and FGF8, resulting in 64% less renal vesicles and 32% fewer nephrons [24]. For the rodent and fetal human kidney, it was demonstrated that the primitive renal vesicle stays connected with the pretubular aggregate [23].

#### The comma-shaped body (Table 1 (no. 4))

For the fetal human kidney, it was shown that a scheduled reduction of comma-shaped bodies takes place between the second to the third semester [25].

#### The S-shaped body (Table 1 (nos. 5 and 6))

In the kidneys of preterm infants who survived for longer, there was no recorded presence of basophilic S-shaped bodies [26]. In the kidneys of premature baboons, it was shown that a decrease in glomerular generations leads to an increase of the renal corpuscle area, including the S-shaped bodies [27].

#### The inner border of the nephrogenic zone (Table 1)

This represents the demarcation line, where the late S-shaped body as the last stage of nephron anlage starts with the maturing process to become a fully functional nephron. In gestational controls, the width of the nephrogenic zone can be up to 150 μm, and in the group of preterm babies, it is significantly smaller at 100 μm [28, 29].

#### The maturing of the nephron (Table 1 (no. 7))

Data regarding the number and spatial distribution (patterning) of maturing glomeruli in the fetal human kidney has to be allocated to the maturation zone [30–32]. This includes glomeruli with a dilated Bowman’s space and a shrunken glomerular tuft [28, 33]. It was published that prematurity not only results in damage of the developing glomeruli, but also causes a decrease in their number [27]. There is no current data regarding atypical development of the tubule portions in the nephron [34].

Data shows that noxae impairing nephrogenesis in the fetal human kidney are not harmful to the nephrogenic niche, the pretubular aggregate, or the renal vesicle (Table 1 (nos. 1–3)). However, the earliest affected stage of the nephron anlage is the S-shaped body (Table 1 (nos. 5–6)) [26, 27]. Interestingly, the effect of harming coincides with a programmed reduction in the comma-shaped bodies (Table 1 (no. 4)) [25]. Both of the stages are passing the process of shaping during early nephron development. This includes specific morphogen signaling, positioning, determination of epithelial segments, spatial expansion, and internal epithelial folding [35]. Although the renal vesicle starts with the shaping process, pathological alterations were reported only for the rodent but not for the fetal human kidney [24]. Since concrete information about metric parameters, spatial positioning, folding, and appearance was lacking, the shaping of a nephron in the fetal human kidney during the phase of late gestation was investigated from a microanatomical perspective.

## Methods

As seen in the figures, specimens of five fetal human kidneys of gestational age between weeks 16 to 18 and later were selected from the stock of preparations, which were used during the Course of Microscopic Anatomy for Medical Students at the University of Regensburg. According to routine methods, the samples of parenchyma were fixed in paraformaldehyde solution and embedded in paraffin wax. Sections of 5 μm thickness were then produced and stained with hematoxylin-eosin solution for analysis by the optical microscope. Screening of stained sections was performed by a Leica DM750 microscope (Leica Microsystems, Wetzlar, Germany). All of the samples were analyzed with a HI Plan 63x/0.75 objective. Images were taken with a Basler Microscopy Pulse 5.0 camera (Basler AG, Ahrensburg, Germany).

More than 3000 images were taken and analyzed for the present investigation. The images presented here are originals, showing one single moment in the process frozen in time. None of the images were altered in order to show the full picture. In order to label the images, they were processed with CorelDRAW X7 (Corel Corporation, Munich, Germany). To obtain information about metric parameters, the images were analyzed with the same program.

## Results

### Structure of the nephrogenic zone

**Fig. 1:**
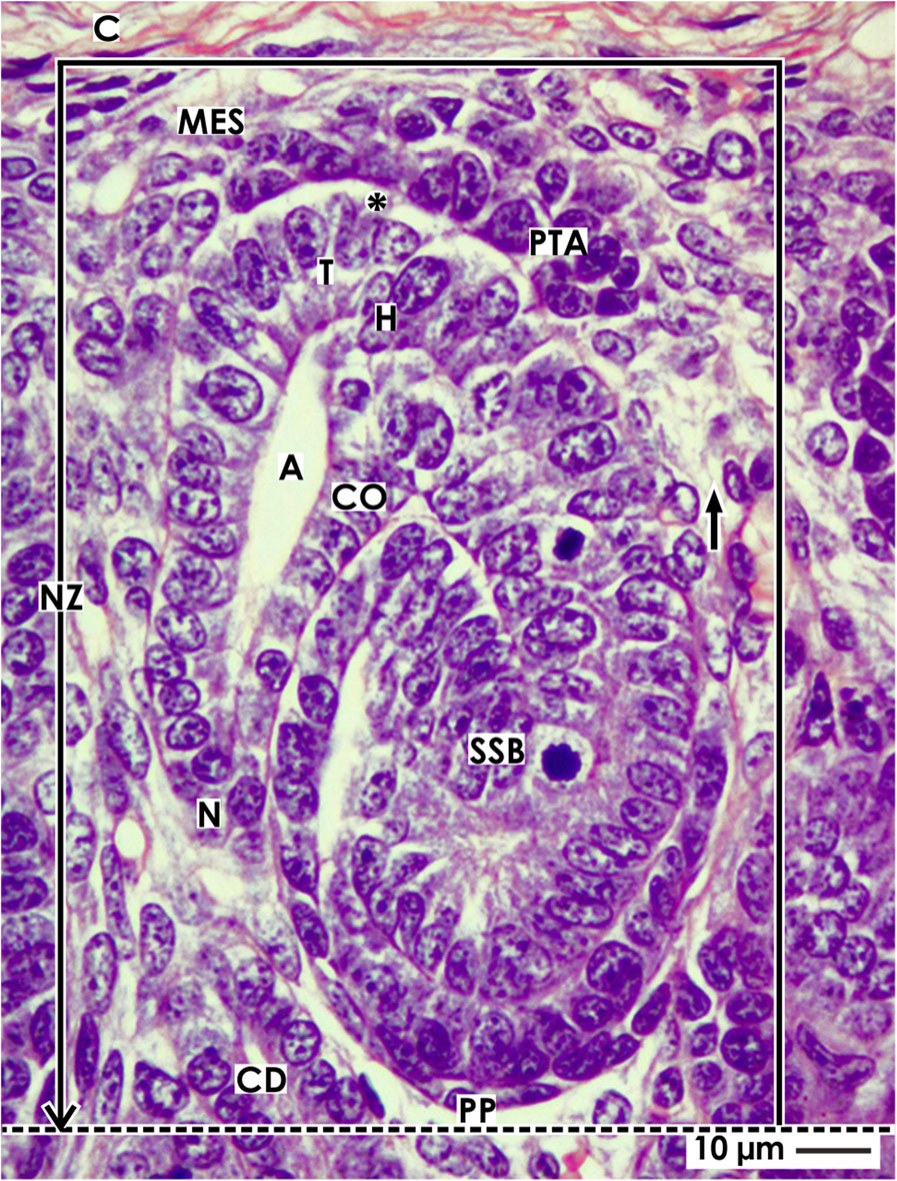
View of a nephrogenic compartment (framed) located in the nephrogenic zone (NZ) of the fetal human kidney by optical microscopy. The outer border is the renal capsule (C). The proximal pole (PP) of an S-shaped body (SSB) represents the inner border (dotted line). The medial border is the distal end of a collecting duct (CD) tubule dilating into a CD ampulla (A). It consists of a tip (T), head (H), conus (CO), and neck (N). The lateral border is a vertically lining perforating radiate artery (short black arrow). Epithelial progenitor cells in the tip of a CD ampulla, an interface (black asterisk), and nephrogenic mesenchymal progenitor cells (MES) indicate a nephrogenic niche. Induced mesenchymal progenitor cells become angular and develop the pretubular aggregate (PTA) along the tip of the CD ampulla

The outer cortex of the fetal human kidney consists of the external nephrogenic zone and the underlying maturation zone (Table 1) [36, 37]. Both extend along the entire surface of each kidney lobe [38, 39]. The outer border of the nephrogenic zone faces the inner side of the renal capsule (Fig. 1), while the inner border lines along the proximal (medulla-orientated) pole of an S-shaped body [28, 29, 40].

**Fig. 2:**
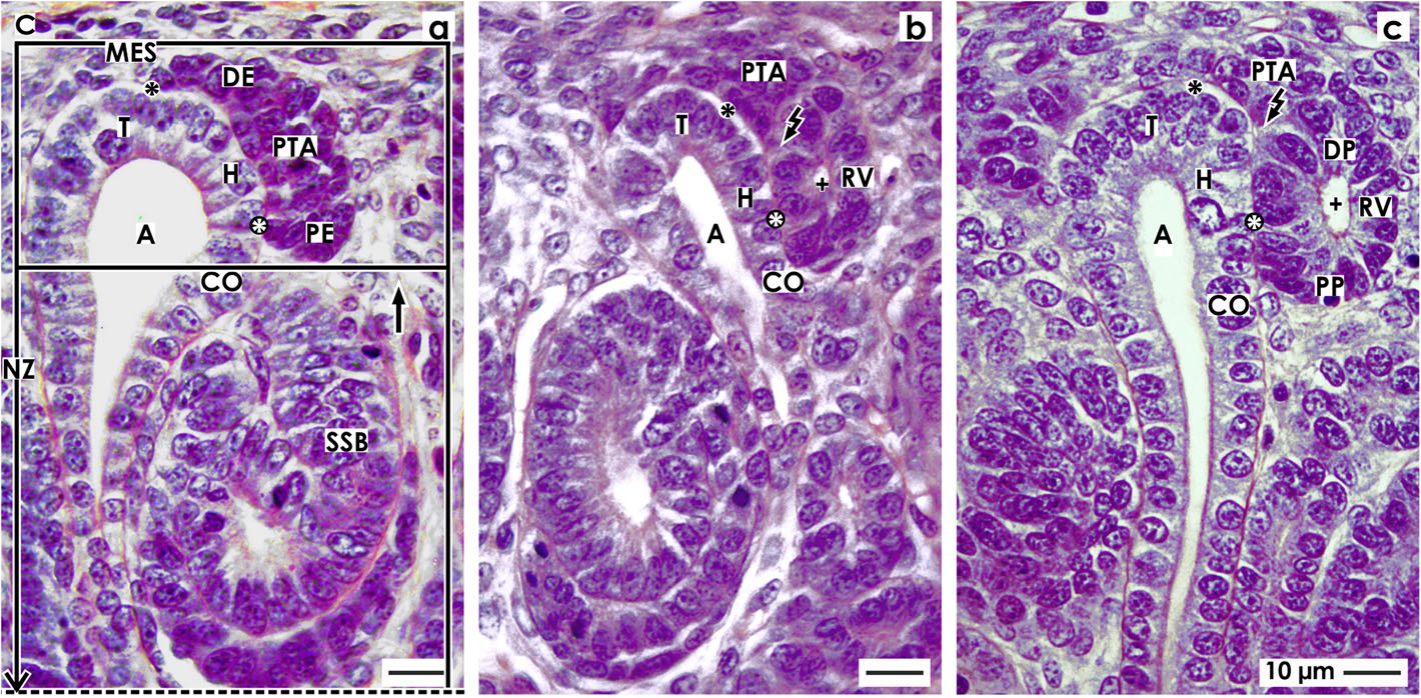
View onto **a** the pretubular aggregate (PTA), **b** the mesenchymal to epithelial transition, and **c** the primitive renal vesicle (RV) in the fetal human kidney by optical microscopy. **a** The upper frame in a nephrogenic compartment marks the district of progenitor cell recruitment. The lower frame shows the area of nephron shaping. The distal end (DE, renal capsule-orientated) of a pretubular aggregate is in contact with nephrogenic mesenchymal (MES) progenitor cells. Separated by a clear interface (black asterisks), it extends along the tip (T) and head (H) of the CD ampulla (A). Between the head and the proximal end (PE, medulla-orientated) of the pretubular aggregate, a close adhesion (white asterisks) is seen. **b** Interstices between cells and smoothening of the surface at the proximal end of the PTA indicate the mesenchymal to epithelial transition. Remarkable is the transition from the clear interface to the close adhesion, and a lumen (cross) becomes visible in the proximal end. The flash indicates the starting separation of the RV from the PTA. **c** A primitive renal vesicle is still part of the pretubular aggregate. At its distal pole (DP), a significant but only partial separation from the PTA is noticed (flash). The epithelium of the renal vesicle contacting the conus (CO) of the CD ampulla is more prominent than at its lateral aspect. The lumen (cross) extends and a basal lamina covers the proximal pole (PP) of the renal vesicle. C renal capsule, short black arrow vertically lining perforating radiate artery, SSB S-shaped body

By drawing vertical lines, the nephrogenic zone can be divided into multiple nephrogenic compartments, which are aligned side by side in a row underneath the renal capsule (Table 1, Fig. 2a). The medial border of a nephrogenic compartment is defined by the vertical course of its neighboring collecting duct (CD) tubule, at the end of which the CD ampulla forms. Meanwhile, the lateral border faces a vertically lining perforating radiate artery. The development of a single nephron from the niche to the late S-shaped body occurs within the nephrogenic compartment. The developmental vector lines perpendicular to the renal capsule and in parallel to the related CD tubule. Each of the nephrogenic compartments can be subdivided by a horizontal line into the above-positioned district of progenitor cell recruitment and the underlying area of nephron shaping.

### District of progenitor cell recruitment

The upper transverse border lines along the inner side of the renal capsule and exhibits a length of about 80 μm (Fig. 2a), while both vertical borders are of about 50 μm in length. However, the lower transverse border crosses between the head and conus of a CD ampulla. At this site, the head of a CD ampulla lies at an outer transverse diameter of 55 μm. Between the tip of a CD ampulla and the inner side of the renal capsule, two to three layers of nephrogenic mesenchymal cells are seen. In the rodent kidney, it was shown that cells of the arising stroma such as fibroblast, hemoendothelial cells, but also macrophages are distributed between them [41].

### Area of nephron shaping

A quadrate with vertical and horizontal lengths of about 100 μm each represents the area of nephron shaping (Fig. 2a), while the upper border lines lying transversely between the head and conus of the CD ampulla. This line meets the upper gusset, where the presumptive connecting tubule (CNT) of a previously developed S-shaped body meets the head of a CD ampulla. As shown later, it continues along the proximal end of a pretubular aggregate and crosses the attachment site of the mature renal vesicle on the CD ampulla. Finally, the line reaches the progenitor cell strand, which is separated during the process of shaping between the proximal end of the pretubular aggregate and the early comma-shaped body. The medial border is formed by the conus and neck of the CD ampulla lining into the CD tubule. The lateral border is near a vertically running perforating radiate artery. When a microscopic specimen is analyzed, the area of nephron shaping shows the attachment of the presumptive CNT on the CD ampulla and either a mature renal vesicle, a comma-shaped body, or an S-shaped body.

### The nephrogenic niche

The targeted meeting between the most inner layer of nephrogenic progenitor cells (Six2^+^/Cited1^+^) and the epithelial progenitor cells integrated in the tip of a CD ampulla represents a nephrogenic niche [23]. The presence of morphogens such as GDNF, Wnts, FGFs, and BMPs initiates the development of a nephron [42, 43]. In the fetal human kidney, one can see that the mesenchymal progenitor cells are separated from the tip of the CD ampulla by a clear interface (Figs. 1 and 2a). After induction, some of the nephrogenic mesenchymal progenitor cells increase in size and become surprisingly angular, the interstices between them enlarge, and then they aggregate along the tip of the CD ampulla forming a pretubular aggregate.

### The pretubular aggregate

It is unclear whether the displacement of induced nephrogenic progenitor cells towards the tip of a CD ampulla occurs by targeted migration or whether they are attracted by the vertically elongating CD ampulla. The subsequent formation of the pretubular aggregate occurs first along the tip and then head of the CD ampulla (Fig. 1 and 2a).

#### Orientation, adhesion, and expansion

During development of an early pretubular aggregate, its distal end (renal capsule-orientated) is connected with the most inner layer of the nephrogenic mesenchymal progenitor cells facing the tip of a CD ampulla (Fig. 2a). By multiplication of cells at the proximal end (medulla-orientated), a transverse broadening of the pretubular aggregate can be seen, while its medial part develops in close contact with the CD ampulla. A clear interface is visible between the distal end of the pretubular aggregate and the tip of the CD ampulla. In contrast, between the proximal end of the pretubular aggregate and the head of the CD ampulla, a close adhesion is noticed. The increased incidence of uneven interstices between cells and smoothening of the surface at the proximal end of the pretubular aggregate indicates the starting polarization.

#### Mesenchymal to epithelial transition

In the proximal part of the mid pretubular aggregate, the mesenchymal to epithelial transition (MET) becomes visible. As seen in the rodent kidney, this process initiates the formation of a renal vesicle [21, 44–46]. However, no comparable conclusions can be made for the fetal human kidney. It can be observed that between the distal and proximal end of the pretubular aggregate, a transverse separation is in progress (Fig. 2b). At a later stage, the development of an epithelium and a small lumen in the proximal part of the pretubular aggregate can be observed.

#### The primitive renal vesicle

The distal pole of the primitive renal vesicle is first connected with the late pretubular aggregate (Fig. 2c). However, at the medial part of the pretubular aggregate and at the point of transition between the clear interface and the close adhesion, a transverse separation line becomes visible. This line then extends towards the middle of the pretubular aggregate, while the lateral part of the pretubular aggregate remains connected with the distal pole of the renal vesicle via a two-layered progenitor cell strand.

Yet, the backdrop is changing. While development up to the primitive renal vesicle occurred in the district of progenitor cell recruitment, the subsequent formation of the extending renal vesicles and comma- and S-shaped bodies takes place in the area of nephron shaping (Fig. 2a).

### Mature, extending, and extended renal vesicles

**Fig. 3:**
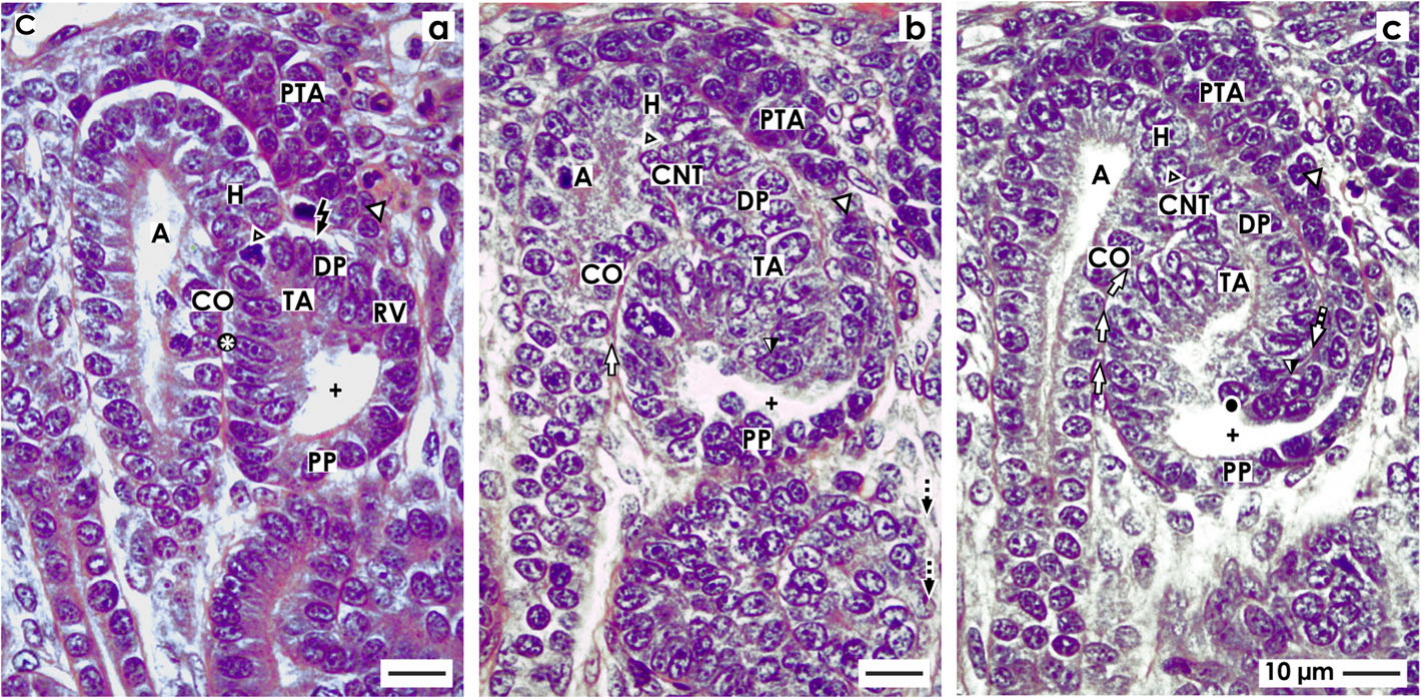
View onto the **a** mature, **b** extending, and **c** extended renal vesicle (RV) in the fetal human kidney by optical microscopy. **a** In a mature renal vesicle, the medial part of the distal pole (DP, small black/white arrow head) is fixed on the CD ampulla (A) at the border between its head (H) and conus (CO). In the middle of the distal pole, the separation (flash) persists. The lateral part of the distal pole remains connected with the pretubular aggregate (PTA) via a two-layered progenitor cell strand (larger black/white arrow head). The proximal pole (PP) is orientated towards the medulla. The lumen (cross) expands unevenly. Between the conus of the CD ampulla and the medial aspect of the renal vesicle, the interphase (white asterisks) lines in vertical direction. **b** The extending renal vesicle is fixed via the future connecting tubule (CNT) on the CD ampulla. At the proximal pole, the lumen is slightly rounded. At the distal pole (DP) the epithelium invaginates (black/white arrow head) to generate the tubulus anlage (TA). The progenitor cell strand (white arrow head) at the lateral part of the distal pole is connected with the PTA. The interphase between the conus of the CD ampulla and the medial aspect of the renal vesicle is transforming into to a vertically lining cleft (white arrows). **c** In the extended renal vesicle, the fixing on the CD ampulla at the border between its head and conus is consolidating by invasion of the future connecting tubule. At the lateral part of the distal pole, the renal vesicle is connected with the pretubular aggregate via a two-layered progenitor cell strand (white arrow head). The epithelium at the distal pole further invaginates towards the underlying lumen, resulting in an inner fold (black spot). The medial leg of the inner fold lines to the part of the CNT that is ending at the head of the CD ampulla. The lateral leg lines to the cell strand (white arrow head) connected with the PTA. Between the legs of the inner fold, a vertically lining cleft (dashed white arrow) arises. The cleft (white arrows) between the medial aspect of the renal vesicle and the conus of the CD ampulla elongates and changes its direction from vertical to slightly transverse. The lumen exhibits a supine C-form. C renal capsule

It has been shown in the rodent kidney that the development of a renal vesicle is unexpectedly complex. At present, a distinction is made between the primitive, mature, and extending renal vesicles 1 to 5 [44, 47]. Immunohistochemistry has revealed that the expansion of the mature and the extended renal vesicle depends on expression of the nectin adaptor protein afadin, which is implicated in the formation of adherens junctions. Comparable data for the fetal human kidney is not available, although in a renal vesicle, the determination of the tubule segments takes place and the process of shaping begins (Fig. 3).

#### Fixing, partial separation, and residual contact

A basic point of reference in a mature renal vesicle is its distal pole. The medial part is fixed onto the CD ampulla at the border between its head and conus (Fig. 3a), the inner part remains separated from the overlying pretubular aggregate, and the lateral part holds up its connection with the pretubular aggregate via a two-layered progenitor cell strand. The contour of the lumen is uneven. Due to starting internal folding of the epithelium, the lumen rounds off at the proximal pole, while at the distal pole, it becomes v-shaped. At the point between the conus of the CD ampulla and the medial aspect of the renal vesicle, a clear interphase becomes visible. This expands in vertical direction and develops towards a cleft. Most notably, the tubule anlage derived from the later CNT elongates in a vertical direction.

#### Primary folding

During development of the extending renal vesicle, the lumen flattens (Fig. 3b). The lumen is still rounded at the proximal pole, while the lumen at the distal pole is restricted due to the fact that the tubule anlage elongates vertically. As the renal vesicle develops further, it begins to widen and lengthen transversely. Now, the clear interface between the conus of the CD ampulla and the medial aspect of the renal vesicle converts further into a vertically lining interstitial cleft. The spatial extension and the arising vertical cleft signal that henceforth the process of folding will determine the further shaping of a renal vesicle. Since a renal vesicle is limited at its proximal pole by the CNT of an earlier established nephron, its future extension is radially directed towards the renal capsule, while in parallel, an elongation of the CD ampulla takes place. Both events are signs for a radial extension of the nephrogenic zone.

#### Secondary folding

In the next phase of shaping, the fixing of the extended renal vesicle onto the CD ampulla at the border between its head and conus is consolidating (Fig. 3c). The later CNT can be seen to invade the epithelium of the CD ampulla. In order to enable this process, the basal lamina of the renal vesicle and the basal lamina of the CD ampulla must be dissolved at the fixing site. The cleft between the medial aspect of the renal vesicle and the conus of the CD ampulla elongates. Near the CNT, it changes the direction from vertical to slightly transverse. This deviation indicates the point at which the renal vesicle begins to wind. The lateral part of the distal pole in an extending renal vesicle is still connected with the pretubular aggregate via the previously mentioned two-layered progenitor cell strand.

Finally, the epithelium of the tubule anlage protrudes vertically to form an inner fold (Fig. 3c). The medial leg of this inner fold lines up with the epithelial cell strand of the later CNT, which ends at the head of the CD ampulla. The tip of the inner fold is positioned in the center of the renal vesicle, while the lateral leg of the inner fold lines up to the progenitor cell strand which is connected to the pretubular aggregate. Between the two legs of the inner fold, a vertically lining cleft is forming. When the lumen takes on a lying C-form, the transition from the extended renal vesicle to the early comma-shaped body occurs.

### The comma-shaped body

**Fig. 4:**
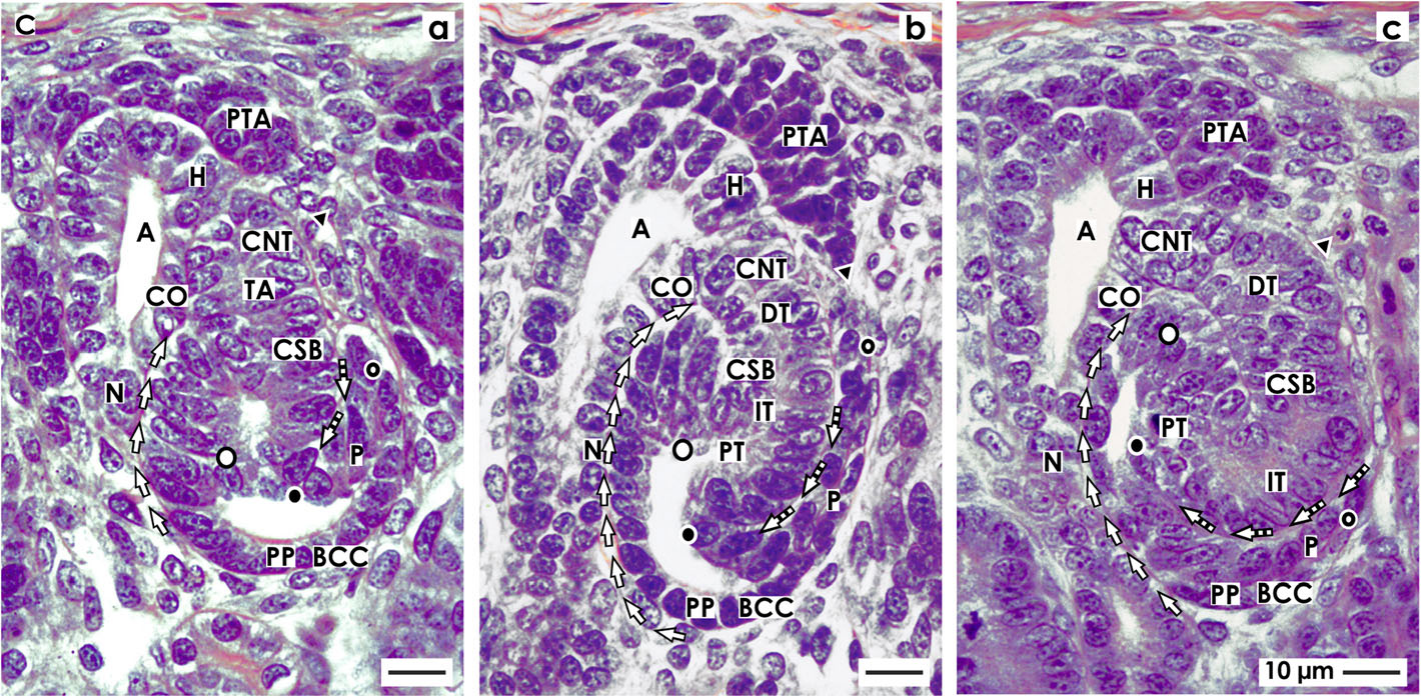
View onto the **a** early, **b** mid, and **c** late comma-shaped body (CSB) in the fetal human kidney by optical microscopy. These are near a CD ampulla (A) along its head (H), conus (CO), and neck (N). The proximal pole (PP) points to the medulla. Here, the parietal (Bowman’s capsule cells, BCC) and the visceral (podocytes, P) cell layers form the glomerulus anlage. Along a vertical axis, the tubule anlage (TA) including the proximal (PT), intermediate (IT), and distal (DT) tubule segments extends. **a** In the early comma-shaped body, the progenitor cell strand (black arrow head) with the pretubular aggregate (PTA) is cut off. The tubule anlage vertically elongates. The cleft (white arrows) between the conus of the CD ampulla and the comma-shaped body opens. Three internal folds become visible. The medial fold (white spot) is near the crotch, where the later connecting tubule (CNT) contacts the conus of the CD ampulla. The tip of this fold is the transition between the parietal cells of Bowman capsule and the proximal tubule. The inner fold (black spot) has a medial leg that lines to the crotch, where the CNT contacts the head of the CD ampulla. Its tip is the transition between the future proximal tubule (medial leg) and the visceral epithelial cell layer (lateral leg) developing into the podocytes (P). The lateral fold (black/white spot) has a medial leg containing the layer of visceral epithelial cells. It is identical with the lateral leg of the inner fold. The tip of the lateral fold lines to the lateral leg containing Bowman capsule cells (BCC). **b** The mid comma-shaped body shows a vertical elongation of the medial, inner, and lateral folds. The cleft (white arrows) between the conus of the CD ampulla and the medial aspect of the comma-shaped body elongates. The cleft (dashed white arrows) at the basal aspect of developing podocytes opens. The Bowman’s capsule cells are at the proximal pole. **c** In the late comma-shaped body, the cleft (white arrows) between the conus of the CD ampulla and the medial aspect of the comma-shaped body is reaching the center of the comma-shaped body. The cleft (dashed white arrows) at the basal aspect of developing podocytes changes course from vertical to transverse. The tubule segments elongate by meandering. C renal capsule

In the rodent and human kidney, the stage-specific expression of proteins in the early comma-shaped body was investigated [47, 48]. The data generated here shows that the progenitor cell strand between the early comma-shaped body and the pretubular aggregate dissolves (Fig. 4a). An internal folding determines the future appearance of the nephron. The close adhesion between the conus of CD ampulla and the medial aspect of the comma-shaped body is replaced by an interstitial cleft that lines vertically in the direction of the future CNT (Fig. 4b). The cleft at the proximal pole of the late comma-shaped body changes the course from vertical to transverse. This indicates a fine positioning of the nephron anlage, subsequent asymmetrical development, and the arising of the glomerulus anlage in the proximal pole (Fig. 4c).

#### Separation and tertiary folding

The extended renal vesicle is connected at its distal pole with the overlying pretubular aggregate via the earlier mentioned two-layered mesenchymal progenitor cell strand (Fig. 3c). Most importantly, during formation of the early comma-shaped body, this connection disappears (Fig. 4a). Henceforth, the comma-shaped body can no longer partake in the progressive recruitment of progenitor cells [23]. At the same time, one can observe that the vertically lining tubule anlage including the future proximal, intermediate, distal, and connecting tubule segments elongates towards the center of the comma-shaped body. This process leads to formation of three internal folds.

1. The medial fold arises near the point, where the epithelial cell strand of the later CNT contacts the conus of the CD ampulla. The tip of the fold is the transition between the later parietal cells of Bowman capsule and the prospective proximal tubule segment (Fig. 4a, b). The folding coincides with elongation of the vertical cleft between the conus of the CD ampulla and the medial aspect of the comma-shaped body.

2. The inner fold has a medial leg that lines up to the point, where the epithelial cell strand of the later CNT contacts the head of the CD ampulla (Fig. 4a, b). The tip of the inner fold is the transition between the prospective proximal tubule segment (medial leg) and the visceral epithelial cell layer (lateral leg) that develops into the podocytes.

3. The lateral fold has a medial leg that contains the layer of visceral epithelial cells developing into the podocytes (Fig. 4a, b). This leg corresponds to the lateral leg of the inner fold. In the lateral leg of the lateral fold, the parietal epithelial cell layer is contained, which develops into the Bowman capsule. As a consequence, the later Bowman’s capsule stretches between the medial leg of the medial fold and the lateral leg of the lateral fold.

#### Consolidation of fixing

During development of the mid comma-shaped body, the connection of its distal pole on the CD ampulla strengthens (Fig. 4b). The epithelium of the later CNT invades the epithelium of the CD ampulla in a cuneiform manner at the border between its head and conus. Furthermore, the comma-shaped body increases its volume by extending vertically. This is paralleled by the elongation of the medial, inner, and lateral folds. It causes the vertical cleft between the conus of the CD ampulla and the medial aspect of the comma-shaped body to increase in length. An elongation is also noticed in the proximal tubule segment and the visceral epithelial cell layer, where the podocytes develop.

#### Exploitation of the available space

During development of the late comma-shaped body, the more or less straight elongation of the tubule segments (Fig. 4a, b) begins to meander (Fig. 4c). The resulting compaction enables an optimal exploitation of the available space within the late comma-shaped body. Furthermore, the cleft between the conus of the CD ampulla and the medial aspect of the comma-shaped body lengthens by winding until it reaches the center of the comma-shaped body. At the proximal pole, an asymmetrical growth can be observed. The cleft between the prospective proximal tubule segment and the visceral epithelial cell layer developing into the podocytes changes direction from vertical to transverse. The opening of this cleft is orientated towards a vertical perforating radiate artery. This positioning enables the invasion of an afferent arteriole towards the establishing glomerular tuft.

### The S-shaped body

The S-shaped body is the last stage of nephron anlage that develops in the nephrogenic zone. Its formation is fluent and was investigated by cell biological methods in the rodent and human kidney [47, 49, 50]. Meanwhile, a distinction is made between the early, mid, and late S-shaped body.

#### Position, orientation, and segmentation

**Fig. 5:**
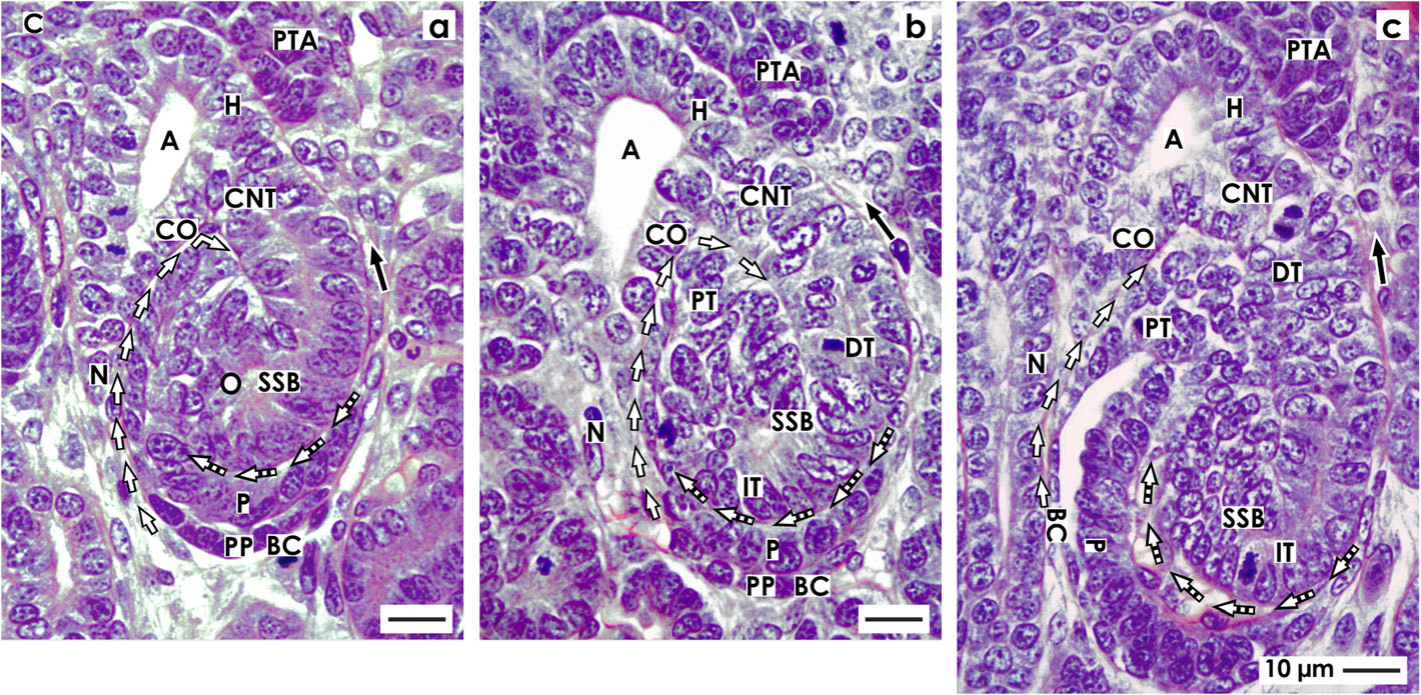
View onto the **a** early, **b** mid, and **c** late S-shaped body (SSB) in the fetal human kidney by optical microscopy. These extend along a CD ampulla (A) between its head (H), conus (CO), and neck (N). The medial aspect is separated from the CD ampulla by a vertical cleft (white arrows). At the distal pole, the connecting tubule (CNT) completes the physiological connection with the CD ampulla. The proximal pole (PP) is orientated towards the medulla. Here, the glomerulus develops including the podocytes (P) and Bowman’s capsule (BC). The proximal (PT), intermediate (IT), and distal (DT) tubule segments develop. **a** The outer cell layer at the proximal pole of an early S-shaped body forms the Bowman’s capsule. Separated by the urinary space in the overlying layer, the podocytes are found. The point of fold between both cell layers is at the deep lateral aspect of the S-shaped body. The transverse cleft (dashed white arrows) above is opening towards a vertically lining perforating radiate artery (short black arrow). **b** At the proximal pole of the mid S-shaped body, cells of the glomerulus differentiate. The Bowman’s capsule becomes flat and the podocytes develop a cobblestone-like appearance. The transverse cleft (dashed white arrow) above opens for vessel formation. At the urinary pole, the proximal tubule (PT) develops a brush border. In the intermediate and distal tubule segments, a lumen becomes visible. **c** The late S-shaped body extends the vertical length. At its proximal pole, the glomerulus is tilting. The Bowman’s capsule is flat and the urinary space is broadening. The facing podocytes increase in number. The cleft (dashed white arrows) at the basal aspect of podocytes changes direction from transverse to vertical. In its interior, the formation of capillaries is noticed. Its opening is orientated towards a vertically lining perforating radiate artery. In the mid and distal third of the S-shaped body, the tubule segments elongate by meandering. C renal capsule

The axis of an early S-shaped body lines perpendicular to the renal capsule (Fig. 5a). At the distal pole, it is connected via the CNT with the CD ampulla. At the proximal pole of an S-shaped body, the parietal epithelial cell layer develops into the Bowman’s capsule. This part represents at the same time the inner border of the nephrogenic zone respectively a nephrogenic compartment (Figs. 1 and 2a). Separated by the future urinary space, the overlying visceral cell layer contains the developing podocytes of the glomerulus (Fig. 5a). The point of fold between the visceral and parietal cell layers can be seen at the lower lateral aspect of the S-shaped body. Above the podocyte layer, a transverse cleft opens laterally towards a vertically lining perforating radiate artery.

The close vicinity of the basal aspect of podocytes, the transverse cleft, and the basal aspect of a tubule segment is remarkable (Fig. 5a). It remains to be answered whether at this site the macula densa of the distal tubule establishes itself and, if so, whether it would begin at this early stage. Within the transverse cleft the glomerular tuft, the intra- and extraglomerular mesangium and the endothelium are forming. Finally, numerous cell divisions in the single tubule segments indicate that an elongation is taking place. The occasional presence of a lumen signals the process of physiological development.

#### Glomerulogenesis and tubule differentiation

At the proximal pole of a mid S-shaped body, the development of the glomerulus is striking due to the fact that it turns towards the conus respectively neck of the related CD ampulla (Fig. 5b). The cells in the parietal layer become flat to establish the Bowman’s capsule, while the cells in the overlying visceral cell layer differentiate into podocytes in a manner similar to a cobblestone arrangement. The overlying transverse cleft extends widthwise. At its opening, an immigration of cells with a bright nucleus is observed. This signals the construction of the glomerular barrier, running mesangiogenesis, and connection with the vertical lining perforating radiate artery via an afferent arteriole. At the urinary pole, an arising brush border indicates that the proximal tubule functionally differentiates. In the distal tubule segment, a lumen becomes visible.

#### Conversion into the maturing nephron

Due to radial expansion of the nephrogenic zone towards the renal capsule, the late S-shaped body extends vertically (Fig. 5c). At its proximal pole, typical morphological signs of the glomerulus become visible. The Bowman’s capsule is seen as a flat epithelial layer. A further broadening of the urinary space is noticed. At the basal aspect of podocytes, capillaries form a network to become part of the arising intraglomerular mesangium. The cleft opening towards a vertically lining perforating radiate artery becomes sickle-shaped, elongates towards the CD ampulla, and changes its course from transverse to vertical. Above the developing glomerulus, in the mid and upper third of the late S-shaped body, numerous cell divisions indicate that the different tubule segments elongate. The occurrence of a lumen and clear cell borders informs us about proceeding functional differentiation. The physiological connection between the CNT and the CD ampulla is completed.

## Discussion

In order to prevent the impairment of nephrogenesis and subsequent oligonephropathy later in life, the search for a suitable therapeutic concept prolonging nephrogenesis in preterm and low birth weight babies has begun [51, 52]. However, a basic prerequisite for the research is to fully understand the damaged stage(s) of nephron anlage, the injured cells, the molecular binding sites of noxae, the altered signal transmission, and the affected metabolic pathways. A special challenge is not only to select eligible drugs, which prolong nephrogenesis, but also to find out a suitable path to administer them in the poorly vascularized nephrogenic zone [53]. Within the last years, a series of viable options have been proposed with regard to aiding regeneration of renal parenchyma [54]. In order to keep our goals realistic, the task for the next decade will be to transfer our current knowledge on the specific development of the nephrogenic zone and the here contained stages of nephron anlage from the rodent kidney to the fetal human kidney [36, 55]. For these reasons, it is understandable that a concept for a reliable prolongation of nephrogenesis is not yet in sight.

In the fetal human kidney, the earliest described point by which noxae has been found to impair nephrogenesis is the S-shaped body (Table 1 (no. 5)) [26]. Most interestingly, the entire loss of basophilic S-shaped bodies by damage coincides with a scheduled reduction of the comma- and S-shaped bodies which occurs between the second and third trimester of pregnancy [25]. It is unknown whether the scheduled reduction, specifically the loss of basophilic S-shaped bodies by noxious stimuli, depends on regulation of genes which controls the number of nephrons or a related epigenetic mechanism [56, 57]. Therefore, it seems probable that the process controlling the scheduled reduction is impeded by noxae impairing nephrogenesis.

When damage of an S-shaped body occurs, it is passing the process of shaping (Figs. 2, 3, 4, and 5). Also, the previously developing comma-shaped body (Table 1 (no. 4)) and renal vesicles (Table 1 (no. 3)) are involved in this basic process [58]. The data shown here provides first insights into this little known and less considered phase of early nephron development in the fetal human kidney during the phase of late gestation. The current illustrations show that a primitive renal vesicle is still part of the pretubular aggregate (Fig. 2b). A step forward in shaping is the partial separation of the mature renal vesicle from the pretubular aggregate, its fixing on the CD ampulla, and the remaining contact via a strand serving the progressive provision of progenitor cells (Fig. 3) [23]. The definitive separation of the early comma-shaped body from the pretubular aggregate occurs at a surprisingly late stage of the process (Fig. 4a). Neither the cellular nor the matricellular or extracellular mechanisms are known, which control the separation. Striking for the further development is the process of internal folding, which occurs between the extended renal vesicle and the comma-shaped body (Figs. 3c and 4a, b). From a microanatomical point of view, this starts with fixing of the renal vesicle on the CD ampulla leading first to the tubule anlage and then to the anlage of the glomerulus (Figs. 4b and 5c).

The data provided informs us about not previously considered morphological details of early nephron development in the fetal human kidney during the phase of late gestation. Consequently, the generated platform can serve to refine the pathological assessment. By analyzing the coordinates and the appearance, it becomes possible to find out whether noxae bind on a specific cell type on the nephron anlage, in the matricellular space, or within the extracellular matrix. Further investigations can be made as to whether noxae interfere with a morphogen receptor, block molecular signaling, or harm a metabolic pathway. Finally, the introduced morphological base serves to investigate aspects of early nephron development previously not taken into consideration by current analytical methods. It is of special importance to identify the molecular mechanisms that determine the first steps of segmentation, pilot the spatial extension of the renal vesicle, master the internal epithelial folding, and control the close cooperation with the CD ampulla.

Unexpectedly, the illustrations shown here touch on the principles of nephron development. It was suggested for the rodent kidney that the entire pretubular aggregate develops into the renal vesicle, the comma-shaped body, and then the S-shaped body [59]. However, for the fetal human kidney during the phase of late gestation, it is here shown that the mature renal vesicle starts with a partial separation from the pretubular aggregate (Figs. 2c and 3a) so that upon extension the renal vesicle remains connected with the pretubular aggregate via a two-layered progenitor cell strand (Fig. 4c). Most importantly, during development of the early comma-shaped body, this progenitor cell strand dissolves (Fig. 4a). At first sight, the recorded separation appears as a more or less unimportant and repeated microanatomical detail. However, at second sight, the separation raises the basic question as to what happens with the remaining pretubular aggregate. Does it disintegrate or does it develop a further primitive renal vesicle at the proximal end? Assuming that a further renal vesicle is formed, a pretubular aggregate including Six2^+^ progenitor cells represents the potential source of a shaping nephron, as previously described in the mouse kidney [60]. In such a case, it is highly conceivable that a pretubular aggregate in the fetal human kidney during the phase of late gestation produces not only one, but successively an unknown number of nephrons.

In Table 1, it can be seen that noxae impairing nephrogenesis leave traces on the stages of nephron anlage. Reflexively, it is assumed that only the contained progenitor cells are harmed. However, it could be just as likely that the mechanism controlling the spatial extension of the renal vesicle (Fig. 2c), interactions with the CD ampulla (Fig. 3a), cleft formation (Fig. 4b), meandering of tubules (Fig. 5a), and the turning of the glomerulus anlage (Fig. 5c) can be targets of noxae. Also, the process of separation between the renal vesicle, namely the early comma-shaped body and the pretubular aggregate, may be vulnerabilities in the development of the fetal human kidney (Figs. 3a and 4a). For example, disturbance of extracellular matrix results in dysregulated matrix metalloproteinases activity, which causes tissue destruction, functional alterations, and local inflammation [61].

## Conclusions

There is evidence that the impairment of nephrogenesis in the fetal human kidney leaves its initial traces on the shaping of the nephron. The data presented here shows that during this developmental phase, basic features such as coordinates, positioning, spatial orientation, and the later appearance of the nephron are determined. Thus, previously not known but crucial points of nephron development suggest that it is necessary to intensify the search for cell and molecular sites impaired by noxae on the stages of nephron anlage by using current biomedical methods.

## Data Availability

The data sets generated and/or analyzed during the current study are not publicly available due to ongoing research but are available from the corresponding author on reasonable request.

## Abbreviations

BMP: Bone morphogenic protein
CITED1: Transcription coactivator
FGF: Fibroblast growth factor
GDNF: Glial derived neurotropic factor
Pax: Pair box protein
Six: Homeodomain transcriptional regulator
Slit2/Robo2: Molecules involved in extra- and intracellular signaling
Spry: Inhibitor of GDNF
Wnt: Wg(wingless)Int-Gen
WT: Wilms tumor protein
